# Structurally driven one-dimensional electron confinement in sub-5-nm graphene nanowrinkles

**DOI:** 10.1038/ncomms9601

**Published:** 2015-10-23

**Authors:** Hyunseob Lim, Jaehoon Jung, Rodney S. Ruoff, Yousoo Kim

**Affiliations:** 1 Surface and Interface Science Laboratory, RIKEN, 2-1 Hirosawa, Wako, Saitama 351-0198, Japan; 2 Center for Multidimensional Carbon Materials, Institute of Basic Science, UNIST-gil 50, Ulsan 689-798, Korea; 3 Department of Chemistry, Ulsan National Institute of Science and Technology (UNIST), UNIST-gil 50, Ulsan 689-798, Korea; 4 Department of Chemistry, University of Ulsan, 93 Daehak-ro, Nam-gu, Ulsan 680-749, Korea

## Abstract

Graphene-based carbon materials such as fullerenes, carbon nanotubes, and graphenes have distinct and unique electronic properties that depend on their dimensionality and geometric structures. Graphene wrinkles with pseudo one-dimensional structures have been observed in a graphene sheet. However, their one-dimensional electronic properties have never been observed because of their large widths. Here we report the unique electronic structure of graphene nanowrinkles in a graphene sheet grown on Ni(111), the width of which was small enough to cause one-dimensional electron confinement. Use of spatially resolved, scanning tunnelling spectroscopy revealed bandgap opening and a one-dimensional van Hove singularity in the graphene nanowrinkles, as well as the chemical potential distribution across the graphene nanowrinkles. This observation allows us to realize a metallic-semiconducting-metallic junction in a single graphene sheet. Our demonstration of one-dimensional electron confinement in graphene provides the novel possibility of controlling its electronic properties not by chemical modification but by ‘mechanical structuring'.

## Abstract (web summary)

Wrinkles in graphene can restrict the motion of electrons to one dimension. Here, the authors observe a one-dimensional van Hove singularity and bandgap opening in a five nanometre wide wrinkle, which enables them to create a metallic-semiconducting-metallic junction in a single graphene sheet.

## Graphical abstract

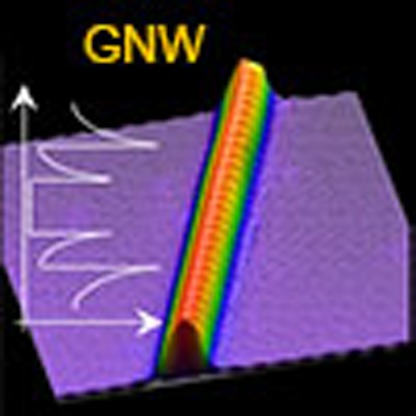

Graphene wrinkles, which are one-dimensional (1D) folded graphene structures, have generally been observed in graphene produced by chemical vapour deposition. These structures have been thought to be the result of the difference in the thermal expansion coefficient between graphene and its substrate^1^. A graphene wrinkle is chemically bonded with surrounding planar epitaxial graphene. Therefore, its unique geometric structure is distinct from those of carbon nanotubes^2^ and graphene nanoribbons^3^,^4^,^5^,^6^, which are indisputably 1D structures. Hence, we define a graphene wrinkle as a ‘pseudo 1D structure' to indicate that it has a 1D shape, but is still a part of a two-dimensional structure.

In the following, we demonstrate the 1D electron confinement in graphene nanowrinkle (GNW) by scanning tunnelling microscopy/spectroscopy (STM/STS), whose width is <5 nm. Moreover, spatially resolved electronic structures have been investigated, and the manipulation of graphene geometry by STM tip has been demonstrated. Our results imply that a semiconducting property can be realized by the mechanical deformation of the graphene geometry not by chemical modification, which is analogous to the case of a strain-induced pseudo magnetic field^7^,^8^ that was discovered in deformed ‘graphene nanobubbles'^7^. The lack of surface functionalization in our approach can prevent the mobility decline due to chemical defects. Moreover, the covalent bonding at the metallic pEG-semiconducting GNW junction can reduce the contact resistance. Our results demonstrate that the interfacial interaction between graphene and the metal substrate provides a novel way to realize a metallic-semiconducting-metallic junction within a single graphene sheet.

## Results

### Structural characterization of GNWs

**Figure 1:**
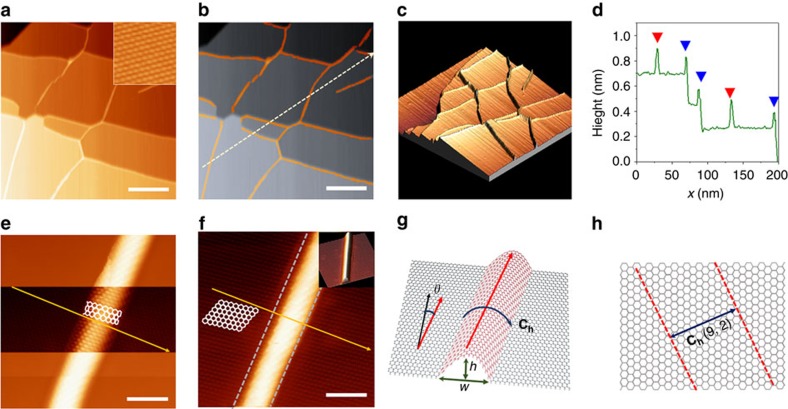
Atomic structure of GNW. (**a**–**c**) STM images of epitaxial graphene on Ni(111). (**a**) Original image, (inset) graphene lattice on pEG region. Scale bar, 40 nm. (**b**) Recoloured image showing GNWs with orange colour. (**c**) Three-dimensional STM image of the epitaxial graphene and GNWs. Scale bar, 40 nm. (**d**) Height profile along the white dashed arrow in **b**. Red triangles and blue triangles indicate the GNWs on the terrace of the Ni(111) surface and at step edges of the Ni(111) substrate, respectively. (**e**,**f**) High-resolution STM images with different scanning conditions. (**e**; top and bottom) *V*_*s*_=1 V and *I*_*f*_=1 nA, (centre) *V*_*s*_=0.05 V and *I*_*f*_=1 nA. Orange arrows indicate the **C**_**h**_ direction identified in **g** and white hexagonal patterns indicate the carbon atoms. (**f**) *V*_*s*_=0.05 V and *I*_*f*_=6 nA. Scale bars, 2 nm. (**g**,**h**) Schematic drawings (**g**) to clarify the meaning of the parameters used to specify the structure of the GNWs and (**h**) for the (9, 2) GNW observed in **e**,**f**.

Epitaxial graphene with GNWs was synthesized by dissociating acetylene on a clean Ni(111) surface. A rapid cooling process is necessary, which is the most critical step to synthesize GNWs. Most of the GNWs were observed in the region where the terrace width of the underlying Ni surface was as small as several tens of nanometres (Fig. 1a–c). These GNWs have been recoloured with orange in Fig. 1b, and a line profile along the white arrow in Fig. 1b is plotted in Fig. 1d, which shows that the GNWs on the terrace have larger widths and lower heights than the GNWs at the step edges. We should note that all GNWs were formed at the step edges (red triangles in Fig. 1d) or propagated from kinks at the step edges (blue triangles in Fig. 1d) of the Ni surface, the implication being that the geometrical structure of the underlying Ni must play a crucial role in the formation of GNWs (Supplementary Figs 1–4; Supplementary Note 1,2).

To analyse the structure of the GNWs in detail, we obtained atomically resolved STM images from an isolated GNW on the terrace under different scanning conditions. The top and bottom regions of the GNW in Fig. 1e were scanned at a sample bias (*V*_*s*_) of 1 V and a feedback current (*I*_*f*_) of 1 nA, whereas the centre region was scanned with a smaller tip–sample distance (*V*_*s*_=0.05 V and *I*_*f*_=1 nA), so that individual carbon atoms could be clearly imaged. Figure 1f was obtained at a much shorter tip–sample distance by increasing *I*_*f*_ up to 6 nA on the same area as Fig. 1e; this clearly shows the graphene lattice of pEG. Both the GNW and pEG areas have the same lattice directions, the indication being that there are no grain boundaries between them. To specify the atomic structure, the height (*h*) and width (*w*) of the GNW were precisely measured. The translation vector (**T**), chiral vector (**C**_**h**_) and chiral angle (*θ*: angle between **T** and the armchair direction) were defined as shown in Fig. 1g. By using the measured values of *w*=2.14 nm, *h*=0.42 nm and *θ*≈10°, we could estimate the indices of the **C**_**h**_ to be (9, 2) for the GNW (Fig. 1e).

### 1D electron confinement in GNW

**Figure 2:**
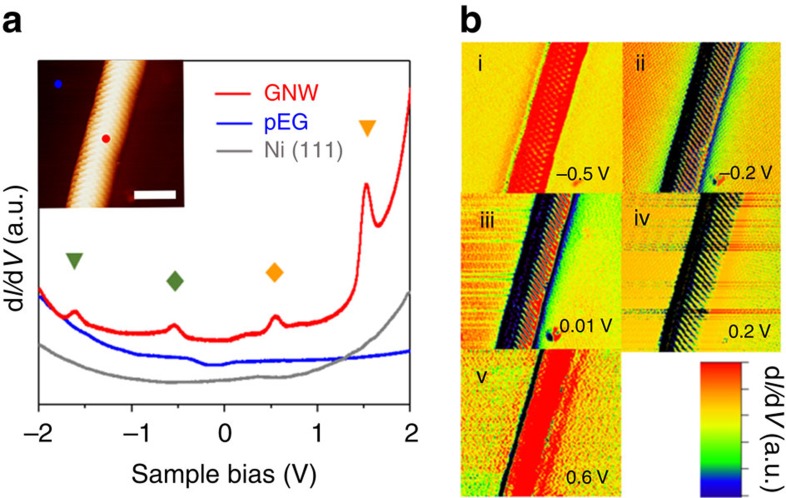
Electronic structures in GNW confirmed by STS. (**a**) d*I/*d*V* spectra on (grey line) bare Ni(111), (blue line) flat graphene, and (red line) GNW as marked in inset STM image. Scale bar, 2 nm. The symbols above the peaks indicate (green diamond) *v*_2_, (green triangle) *v*_1_, (orange triangle) *c*_1_ (from conduction band) and (orange diamond) *c*_2_. (**b**) d*I/*d*V* mapping images of GNWs obtained at *V*_*s*_=(i) –0.5 V, (ii) –0.2 V, (iii) 0.01 V, (iv) 0.2 V and (v) 0.6 V.

The electronic structures of GNWs located on the terraces of the underlying Ni(111) were investigated by STS and d*I/*d*V* mapping. The d*I/*d*V* spectrum measured on the GNW (red line in Fig. 2a) by STS measurement included four strong peaks, *v*_1_ and *v*_2_ for the valence band side, and *c*_1_ and *c*_2_ for the conduction band side, the indication being that there were discrete electronic states in the local density of states (LDOS); this behaviour is similar to the ‘band-gap opening' feature observed in a single-wall carbon nanotube (SWCNT)^9^,^10^,^11^. There was no evidence of such discrete electronic states on the pEG area (blue line in Fig. 2a). The Dirac point was not clearly apparent, even on the pEG area, because chemisorption on a Ni substrate strongly perturbs the electronic structure of graphene^12^,^13^,^14^,^15^. The d*I/*d*V* images in Fig. 2b revealed the spatial distribution of the LDOS, which were obtained at various *V*_*s*_, including the voltages at peak positions (*V*_*s*_=–0.6 V (*v*_1_) and 0.5 V (*c*_1_)) and between *v*_1_ and *c*_1_ (*V*_*s*_=–0.2, 0.01 and 0.2 V) as well At the peak positions, the measured d*I/*d*V* intensities, which are indicative of the LDOS, were higher on the GNW area than on the pEG area. In contrast, lower LDOSs were observed on the GNW at *V*_*s*_ values in the energy gap between *v*_1_ and *c*_1_ (*ΔE*_*g*_) (Supplementary Fig. 5 for d*I/*d*V* images at the second set of peaks (*V*_*s*_=−1.6 V (*v*_2_) and 1.5 V (*c*_2_)), and *V*_*s*_ between the first and second peaks (*V*_*s*_=−1.0 V and 1.0 V)). The d*I/*d*V* images clearly show the metallic-semiconducting-metallic junction across a GNW, which has never been observed in the d*I/*d*V* spectra previously measured on other graphene wrinkles^6^,^16^.

We considered two plausible origins of these peaks, a strain-induced magnetic field and a 1D electron confinement. An induced magnetic field due to the high curvature in graphene can result in Landau levels in the graphene, as observed in graphene nanobubbles^7^. 1D electron confinement can also result in discrete states (van Hove singularities (vHS)) in electronic structures, a phenomenon that has been extensively investigated in SWCNTs with various chiralities^2^,^9^,^10^,^11^,^17^. To check the first possibility, that is, pseudo-magnetic-field based mechanism, the d*I/*d*V* spectra in the present work were compared with Landau levels observed in monolayer graphene under magnetic fields^18^,^19^, as well as ones observed in graphene nanobubbles^7^. In a monolayer graphene sheet, zeroth Landau level (*n*=0) should be observed near Fermi level (*E*_*F*_), since electrons behave like a Dirac fermion. In addition, the position of the *n*th Landau level (*E*_*n*_) follows the relationship 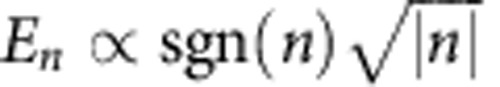 (ref. 7). In our work, however, discrete energy levels (*v*_1_ or *c*_1_) are too far from the *E*_*F*_ to assign one of them as the zeroth Landau level, even when considering the doping effect. Also, the energy gaps between the peaks do not match the relationship, 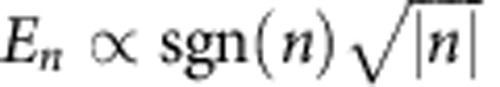. Therefore, we have excluded the pseudo-magnetic-field based mechanism for our results.

**Figure 3:**
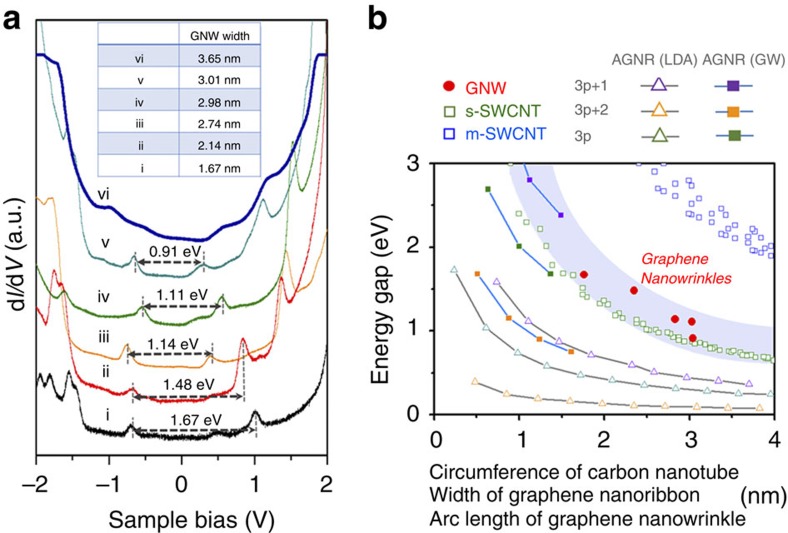
Width-dependent STS studies in GNWs. (**a**; i–vi) d*I/*d*V* spectra obtained from various GNWs with different widths. (**b**) Arc-length dependence of energy gap in GNWs, and comparison with calculated values of the peak differences in the SWCNTs and energy gaps in the AGNR. The calculated values for the SWCNTs, AGNR(LDA) and AGNR(GW) were adapted from Kataura *et al*.^17^, Son *et al*.^20^ and Yang *et al*.^21^, respectively.

To examine the plausibility of the second possible mechanism, that is, 1D electron confinement, we investigated the width dependence on *ΔE*_*g*_, which should show a relationship to *w* (*ΔE*_*g*_∝1/*w*), if *ΔE*_*g*_ originates from 1D electron confinement. Figure 3a shows the d*I/*d*V* spectra obtained from the other GNWs within the width range 1.67 nm (a) to 3.65 nm (f; Supplementary Fig. 6). It is apparent that *ΔE*_*g*_ values decreased with increasing widths of the GNWs, but vHS peaks were not observed in GNWs with widths >3.5 nm; note that we tested 26 GNWs, the distribution of which is provided in Supplementary Fig. 7. The width dependence implies that 1D electron confinement is the most plausible explanation for the unique electronic structures of GNWs. To strengthen the 1D electron confinement mechanism, we compared our results with the electronic structures of a SWCNT and a graphene nanoribbon, archetypal 1D carbon materials showing 1D electron confinement phenomena. For a reliable comparison, the actual length for electron confinement (*L*) is used rather than width of the GNW or the diameter of the SWCNT, because *L* is more directly related to *ΔE*_*g*_ induced by 1D electron confinement. Hence, *ΔE*_*g*_s measured in GNWs, and calculated *ΔE*_*g*_s for both SWNCTs^17^ and an armchair graphene nanoribbon (AGNR)^20^,^21^ are plotted in Fig. 3b as a function of *L*, that is, arc length of GNW, circumference of SWCNT and width of AGNR, respectively. The arc length of the GNW was estimated on the assumption that the GNW arc shape of its cross section is a part of a circle (Supplementary Figs 8–10; Supplementary Table 1; Supplementary Note 3). The values for SWCNTs are extracted from the Kataura Plot^17^, which are calculated by the tight-binding method, and the band gaps of AGNR are estimated by two methods, the local density approximation (LDA)^20^ and the GW approximation^21^, that are used for comparison. Incidentally, LDA usually gives underestimated energy values, while GW values for AGNR are expected to be much closer to reality^21^. Figure 3b shows that the *ΔE*_*g*_s of the GNWs are comparable to the variation of *ΔE*_*g*_s in AGNR and semiconducting SWCNT, which supports the idea that 1D electron confinement in GNWs is more convincing than a pseudo-magnetic-field based mechanism. However, it is noteworthy that circular boundary conditions in the SWCNT impose an even number of nodes on the wave function along the circumference vector, while the number of nodes can be either even or odd in both GNW and GNR. We therefore believe that the comparison of GNW with GNR is appropriate enough to rationalize our work. However, further studies would be required not only to fully understand the correlation between *ΔE*_*g*_ and arc length, but also to reveal the other effects on *ΔE*_*g*_, such as curvature and chirality of the GNW.

### Origin of 1D electron confinement in pseudo 1D structure

Although the width dependence verifies the 1D electron confinement in GNWs, it is simply not understood how GNWs connected with pEG (pseudo one dimensionality) can have the 1D electronic structure. The strong interaction between pEG and the Ni(111) substrate may play a critical role in making a 1D electron channel along GNWs. Owing to the strong interaction, the *π* orbital of the pEG is hybridized with the *d* orbital of the Ni surface (*π*–*d* hybridization)^13^,^14^,^15^,^22^, while pure *π* orbitals remain at the GNW owing to the freestanding feature of GNW from the Ni surface (Supplementary Fig. 11a). These different degrees of interaction at the interface between pEG and GNW isolate the electronic structure of GNW, which provides the 1D ballistic electron channel along the GNW. It has already been suggested that the spatially different degree of interaction with the substrate is sufficient for inducing local electron confinement in a graphene^23^,^24^. Angle-resolved photoemission spectroscopy study has demonstrated the opened bandgap in the graphene sheet on the sidewall facet of a SiC substrate^23^. The origin of the local bandgap was explained with the nanoscale electron confinement, which is due to the fact that the nanoscale graphene has non-interacting parts on the sidewall between the graphene-pinned SiC (0001) surface^24^.

Tao *et al*.^6^ observed a folded graphene structure at the edges of graphene nanoribbons deposited on Au(111), the dimension of which is similar to those of GNWs observed in this study. Nevertheless, vHS peaks induced by 1D electron confinement did not appear, while edge-state peaks appeared near *E*_*F*_ in the d*I/*d*V* spectra. This implies that the strong interaction at the pEG is essential for the 1D electron confinement. Because the graphene nanoribbons were physically deposited by a spin-coating method on a Au(111) substrate^6^, the interaction between the graphene nanoribbons and the substrate would have been relatively weak compared with the interaction between the pEGs and Ni(111) in our studies, which might be insufficient for isolating an electronic structure at the folded graphene region.

### Spatially resolved-STS studies across GNW

**Figure 4:**
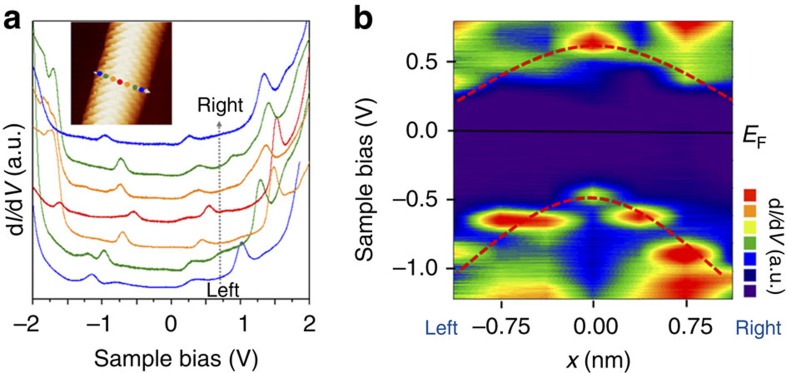
Spatially resolved-STS studies across GNW. (**a**) d*I/*d*V* spectra measured along the line perpendicular to the GNW direction. Coloured dots in the inset of STM images indicate the positions where the d*I/*d*V* spectra were obtained by STS measurement. (**b**) Spatially resolved-STS map from **a** with *V*_*s*_ ranging from –1.3 to 0.7 V. Red dotted lines show the behaviour of changes in *v*_1_ and *c*_1_ with respect to their spatial positions.

We also investigated changes of electronic structure in a direction perpendicular to the GNW. Spectra of d*I/*d*V* were measured at different positions across the GNW (Fig. 4a). It is noteworthy that we could not measure the d*I/*d*V* spectrum at the boundary position, because the tunnelling current became unstable when the STM tip was close to the boundary. These spectra were converted to a spatially resolved-STS map (Fig. 4b) within the *V*_*s*_ range −1.3 to 0.7 V. The spatially resolved-STS map revealed that the positions of *v*_1_ and *c*_1_ were shifted along the **C**_**h**_ direction, the indication being that the chemical potential was different. Compared with the centre position of the GNW, the *E*_*F*_s to both the left and right of the centre position were closer to *c*_1_, the implication being that the chemical potential was larger in the GNW than in the pEG area. We suggest that the asymmetry of the spatially resolved-STS on the left and right sides may be attributed to the asymmetric geometry of the tip. Based on first principles, electron transfer from Ni to epitaxial graphene has been predicted at the epitaxial graphene–Ni contact region^13^,^22^. The pEG region interacts strongly with the Ni substrate, the result being electron transfer from the substrate, whereas the GNW is less charged due to the negligible degree of interaction with its substrate. These different degrees of electron transfer determine the electric field across the GNW and may induce the parabolic distribution of *c*_1_ and *v*_1_ in the spatially resolved-STS (Supplementary Fig. 11b).

### Manipulation of the GNW structure by the STM tip

**Figure 5:**
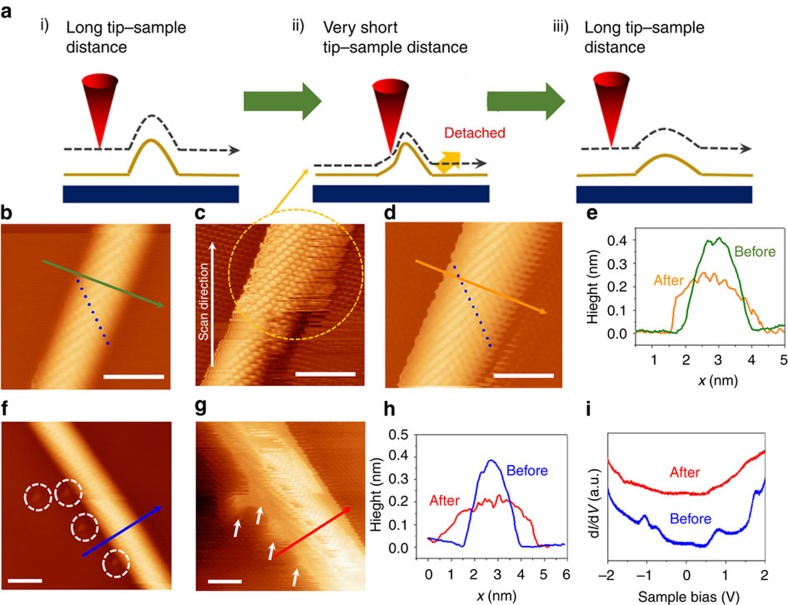
Manipulation of GNW structure by STM. (**a**) Schematic illustration of tip-induced manipulation of GNW. (**b**–**d**) STM images of GNWs with different scanning conditions for the manipulation. (**b**) Before the manipulation obtained with *V*_*s*_=0.01 V and *I*_*f*_=6 nA. Scale bar, 2 nm. (**c**) The manipulation process with *V*_*s*_=0.05 V and *I*_*f*_=6 nA. Scale bar, 2 nm. (**d**) After the manipulation obtained with *V*_*s*_=0.01 V and *I*_*f*_=6 nA. Scale bar, 2 nm. Blue dots in **b** and **d** indicate the positions of carbon atoms along zigzag direction. They reveal the increased number of carbon atoms along zigzag direction of GNW. (**e**) Height profiles before (**b**) and after (**d**) the manipulation. (**f**–**i**) Another example of the manipulation showing a substrate defect effect. (**f**,**g**) STM images before and after reaction. Scale bars, 2 nm. White-dotted circles in **f** indicate the substrate defects, and white arrows in **g** indicate the same positions after the manipulation. (**h**) Height profiles for (**f**,**g**). (**i**) d*I/*d*V* spectra before (**f**) and after (**g**) manipulation.

Finally, we attempted to manipulate the GNW by the STM tip (Fig. 5a). An STM image of Fig. 5b was obtained with *V*_*s*_=0.05 V and *I*_*f*_=6 nA. When the tip was moved closer to the GNW (*V*_*s*_=0.01 V and *I*_*f*_=6 nA), the STM image became very unstable (scratched lines appeared), and the width of the GNW became broader (Fig. 5c). Subsequently, Fig. 5d was obtained with the same scanning conditions used to obtain Fig. 5b, and the larger width and smaller height of the GNW were confirmed from the STM image (Fig. 5e). To explain these phenomena, we suggested a tip-induced manipulation mechanism (Fig. 5a). The mechanical force induced by the short distance between the tip and the GNW caused high strain in the GNW. If the induced strain was high enough to overcome the interaction between the pEG and substrate (Fig. 5a (ii)), the pEG beside the GNW can become detached. The result is a larger width and smaller height of the GNW. Figure 5f–i show another example of the manipulation. Several defects are observed near the GNW before the manipulation as marked with white-dotted circles in Fig. 5f. During the manipulation, the detachment of graphene from the underlying Ni substrate more easily occurred on these defects than the other region due to the weak interaction at these points, which resulted in the local deformation of GNW structure as shown in Fig. 5g. The electronic structures before and after the manipulation have been also investigated by STS measurements (Fig. 5i). The pre-existing vHS disappeared in the d*I/*d*V* spectrum after the manipulation, which is possibly because the manipulation caused increase of the GNW width larger than the width-limitation (∼3.5 nm) corresponding the threshold width showing vHS. Another possibility that cannot be excluded is possible modification of the tip apex during the manipulation that can affect the spectral shape to some extent.

## Discussion

Our results constitute the demonstration of electron confinement in GNWs with dimensions <5 nm and the demonstration of an open bandgap, which implies that GNWs have semiconducting properties. Many efforts have been made to manipulate the electronic properties of graphene by chemical modification^25^,^26^. In contrast, our study shows a new possibility of controlling the electronic structure of graphene by engineering its geometric structure. Compared with chemical approaches, our approach does not involve the breaking of chemical bonds in graphene layers, which causes the carrier mobility to decrease. Moreover, the metallic pEG and semiconducting GNW are covalently bonded, and their work function should be similar. We therefore suggest that the interface between pEG and GWN will provide Ohmic-like behaviour in electron transport with very low contact resistance (Supplementary Fig. 12). This concept of controlled mechanical deformation does not result in new chemical species but rather in new forms of graphene-based carbons, which consist of only carbon atoms.

## Methods

### Sample preparation and characterization by STM

The experiments were performed in an ultrahigh-vacuum, low-temperature STM (Omicron LT-STM) at 4.7 K. The Ni(111) single crystal was cleaned by repetitive Ar^+^ sputtering-and-annealing cycles. EGs consisting of GNWs were synthesized in a ultrahigh-vacuum chamber (base pressure: 2 × 10^–7^ Pa) by dissociating C_2_H_2_ (
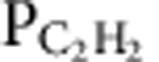
:2 × 10^–5^ Pa) on Ni(111) at 650 °C for 5 min. For rapidly cooling, the sample was immediately removed from the heating stage after the reaction. All STM images were obtained in a constant-current mode. We measured the differential conductance (d*I/*d*V*) with a lock-in amplifier by applying an a.c. bias of 50 mV (797 Hz) to the tunnelling bias. We focused only on the GNWs on the terrace of the Ni(111) surface. A total of 27 individual GNWs were observed with atomically resolved STM images.

## Additional information

**How to cite this article:** Lim, H. *et al*. Structurally driven one-dimensional electron confinement in sub-5-nm graphene nanowrinkles. *Nat. Commun.* 6:8601 doi: 10.1038/ncomms9601 (2015).

## Supplementary Material

Supplementary Information Supplementary Figures 1-12, Supplementary Table 1, Supplementary Notes 1-3 and Supplementary References

## References

[b1] Li X. . Large-area synthesis of high-quality and uniform graphene films on copper foils. Science 324, 1312–1314 (2009). 19423775 10.1126/science.1171245

[b2] Saito R., Dresselhaus G. & Dresselhaus M. S. Physical Properties of Carbon Nanotubes Imperial College Press (1998).

[b3] Han M. Y., Özyilmaz B., Zhang Y. & Kim P. Energy band-gap engineering of graphene nanoribbons. Phys. Rev. Lett. 98, 206805 (2007). 10.1103/PhysRevLett.98.206805 17677729

[b4] Li X. . Chemically derived, ultrasmooth graphene nanoribbon semiconductors. Science 319, 1229–1232 (2008). 18218865 10.1126/science.1150878

[b5] Wang X. . Room-temperature all-semiconducting sub-10-nm graphene nanoribbon field-effect transistors. Phys. Rev. Lett. 100, 206803 (2008). 18518566 10.1103/PhysRevLett.100.206803

[b6] Tao C. . Spatially resolving edge states of chiral graphene nanoribbons. Nat. Phys. 7, 616–620 (2011).

[b7] Levy N. . Strain-induced pseudo–magnetic fields greater than 300 tesla in graphene nanobubbles. Science 329, 544–547 (2010). 20671183 10.1126/science.1191700

[b8] Guinea F., Katsnelson M. I. & Geim A. K. Energy gaps and a zero-field quantum hall effect in graphene by strain engineering. Nat. Phys. 6, 30–33 (2010).

[b9] Wilder J. W. G. . Electronic structure of atomically resolved carbon nanotubes. Nature 391, 59–62 (1998).

[b10] Shin H.-J., Clair S., Kim Y. & Kawai M. Substrate-induced array of quantum dots in a single-walled carbon nanotube. Nat. Nanotechnol. 4, 567–570 (2009). 19734928 10.1038/nnano.2009.182

[b11] Clair S., Kim Y. & Kawai M. Step-edge faceting and local metallization of a single-wall semiconducting carbon nanotube. J. Appl. Phys. 110, 073710 (2011).

[b12] Batzill M. The surface science of graphene: metal interfaces, CVD synthesis, nanoribbons, chemical modifications, and defects. Surf. Sci. Rep. 67, 83–115 (2012).

[b13] Khomyakov P. A. . First-principles study of the interaction and charge transfer between graphene and metals. Phys. Rev. B 79, 195425 (2009).

[b14] Varykhalov A. . Electronic and magnetic properties of quasifreestanding graphene on ni. Phys. Rev. Lett. 101, 157601 (2008). 18999644 10.1103/PhysRevLett.101.157601

[b15] Dedkov Y. S., Fonin M., Rüdiger U. & Laubschat C. Rashba effect in the graphene/Ni(111) system. Phys. Rev. Lett. 100, 107602 (2008). 18352231 10.1103/PhysRevLett.100.107602

[b16] Guo Y. & Guo W. Electronic and field emission properties of wrinkled graphene. J. Phys. Chem. C 117, 692–696 (2012).

[b17] Kataura H. . Optical properties of single-wall carbon nanotubes. Synth. Met. 103, 2555–2558 (1999).

[b18] Novoselov K. S. . Two-dimensional gas of massless dirac fermions in graphene. Nature 438, 197–200 (2005). 16281030 10.1038/nature04233

[b19] Zhang Y., Tan Y.-W., Stormer H. L. & Kim P. Experimental observation of the quantum hall effect and berry's phase in graphene. Nature 438, 201–204 (2005). 16281031 10.1038/nature04235

[b20] Son Y.-W., Cohen M. L. & Louie S. G. Energy gaps in graphene nanoribbons. Phys. Rev. Lett. 97, 216803 (2006). 17155765 10.1103/PhysRevLett.97.216803

[b21] Yang L. . Quasiparticle energies and band gaps in graphene nanoribbons. Phys. Rev. Lett. 99, 186801 (2007). 17995426 10.1103/PhysRevLett.99.186801

[b22] Giovannetti G. . Doping graphene with metal contacts. Phys. Rev. Lett. 101, 026803 (2008). 18764212 10.1103/PhysRevLett.101.026803

[b23] Hicks J. . A wide-bandgap metal-semiconductor-metal nanostructure made entirely from graphene. Nat. Phys. 9, 49–54 (2013).

[b24] Palacio I. . Atomic structure of epitaxial graphene sidewall nanoribbons: flat graphene, miniribbons, and the confinement gap. Nano Lett. 15, 182–189 (2015). 25457853 10.1021/nl503352v

[b25] Liu J., Tang J. & Gooding J. J. Strategies for chemical modification of graphene and applications of chemically modified graphene. J. Mater. Chem. 22, 12435–12452 (2012).

[b26] Bekyarova E. . Advances in the chemical modification of epitaxial graphene. J. Phys. D 45, 154009 (2012).

