# Supplementary material for: Structurally driven one-dimensional electron confinement in sub-5-nm graphene nanowrinkles
Source: Nat Commun. 2015 Oct 23;6:8601. doi: 10.1038/ncomms9601 (PMC4639805; doi:10.1038/ncomms9601)
Supplement: Supplementary Information — Supplementary Figures 1-12, Supplementary Table 1, Supplementary Notes 1-3 and Supplementary References [file ncomms9601-s1.pdf]

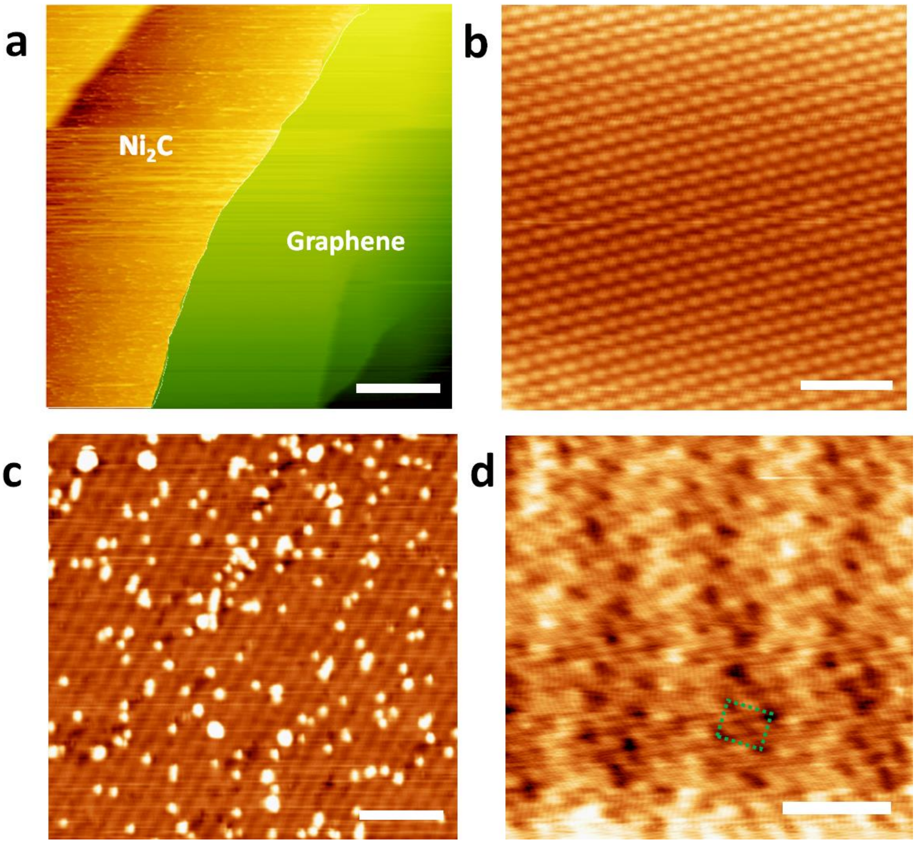

Supplementary Figure 1 | STM images of graphene and Ni_2C. **a**, STM image near the edge of an EG. Scale bar, 150 nm. **b,** Atomically resolved STM image on the planar EG region. Scale bar, 1 nm. **c**, STM image outside of the EG confirming the Ni_2C line structures and contaminants. Scale bar, 8 nm. **d**, Atomically resolved STM image of Ni_2C surface structure. Scale bar, 1 nm.

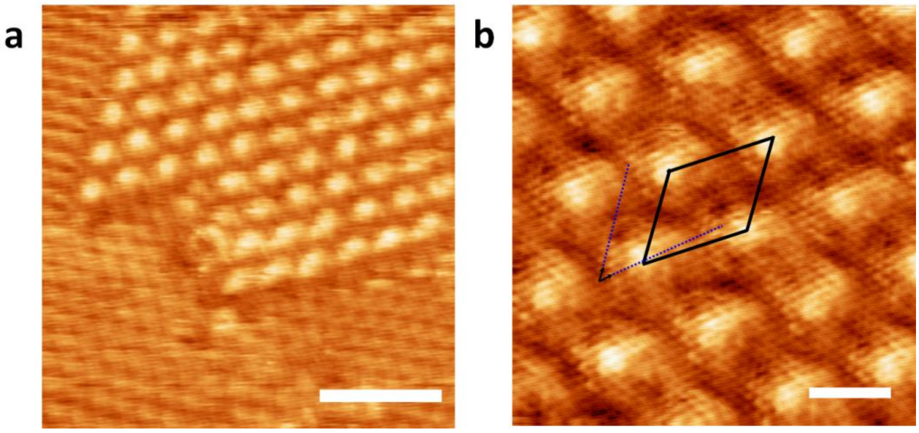

Supplementary Figure 2 | Moiré patterns at mis-oriented region. a,**b**, STM images of Moiré pattern. Scale bars, 5 nm and 2 nm, respectively. In Supplementary Fig. 2b, the atomic lattice of EG can be seen. The black rhombus and blue dotted arrows indicate the Moiré unit cell and EG unit vector directions, respectively.

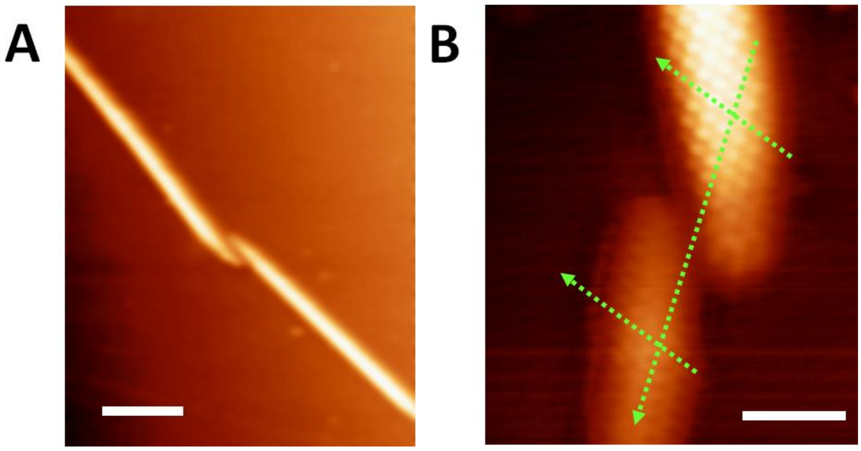

Supplementary Figure 3 | Junction structure of GNWs. a, STM image of a junction between GNWs. Scale bar, 8 nm. **b**, atomically resolved STM image obtained from the other junction. Scale bar, 2 nm. Green dotted lines indicate the zigzag directions of the honeycomb lattice. The lattice directions in the two GNWs are the same, the implication being that the two GNWs are parts of one graphene sheet.

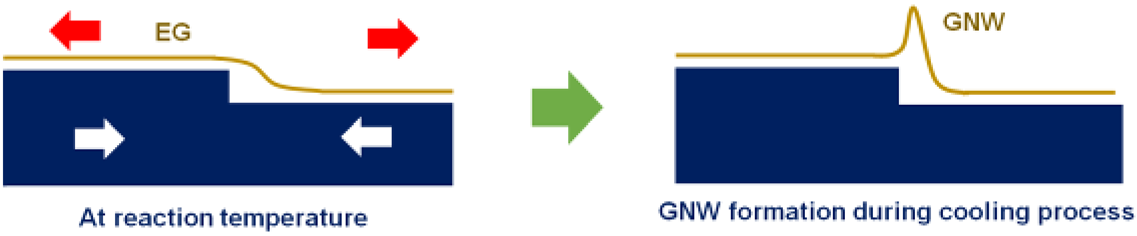

Supplementary Figure 4 | Schematic illustration of the mechanism of formation of a GNW.

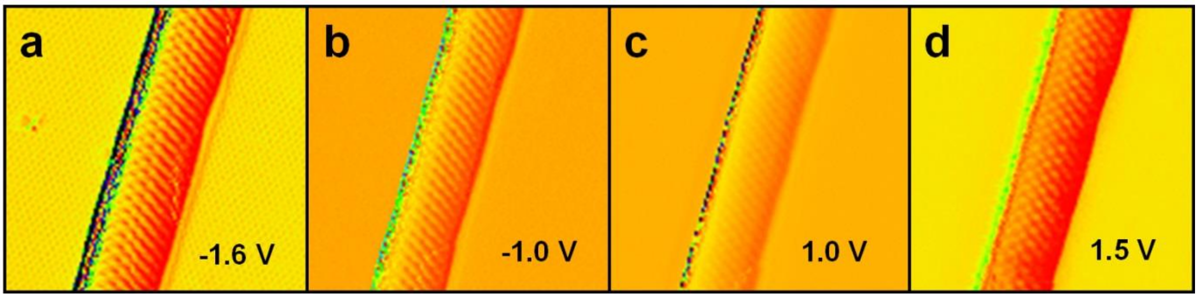

Supplementary Figure 5 | *dI/dV* mapping images of GNWs for second peaks (c_1 and c_2). **a**, V_s = –1.6 V (v_1), **b**, –1.0 V, **c** 1.0 V, **d**, 1.5 V(c_2).

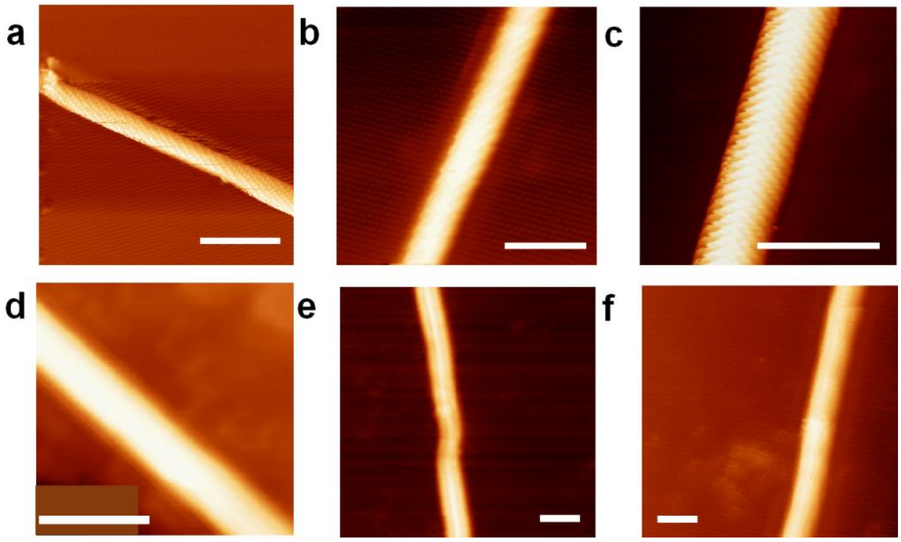

Supplementary Figure 6 | STM images of GNWs. the STS spectra of which are plotted in Fig. 3a. All scale bars, 4 nm.

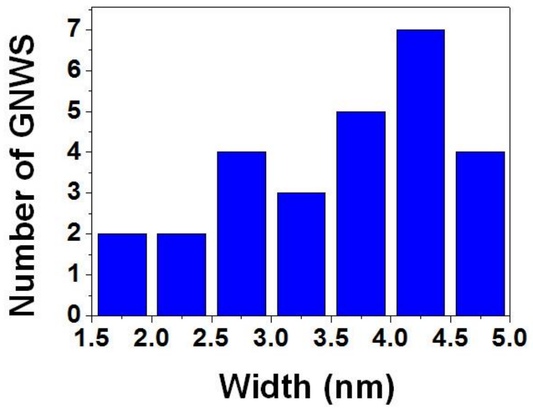

Supplementary Figure 7 | Width-distribution confirmed in the experiment from a total of 27 GNWs.

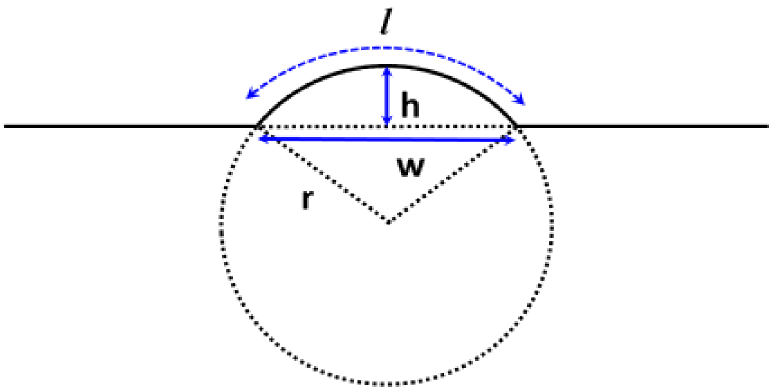

Supplementary Figure 8 | Schematic image showing the relationship between, *l* vs. *h*, *w*.

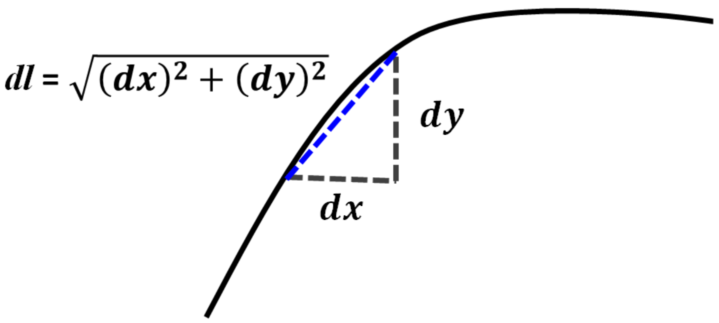

Supplementary Figure 9 | Schematic image showing the relation, *dl* vs. *dx* and *dy*.

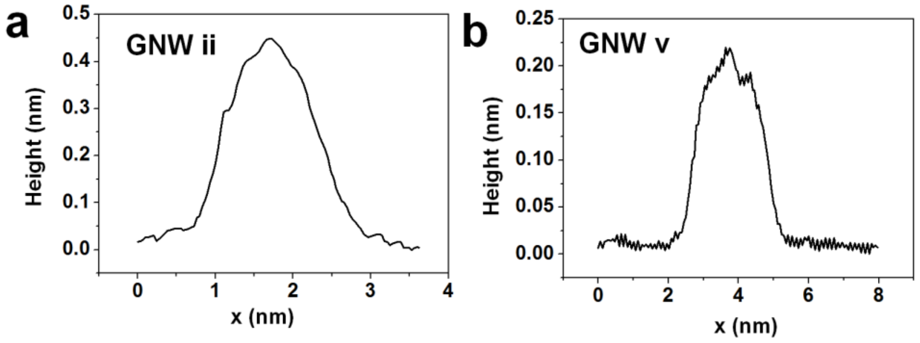

Supplementary Figure 10 | Height profiles for GNW ii and v. a, GNW ii with smooth height profile. b, GNW v with rougher height profile.

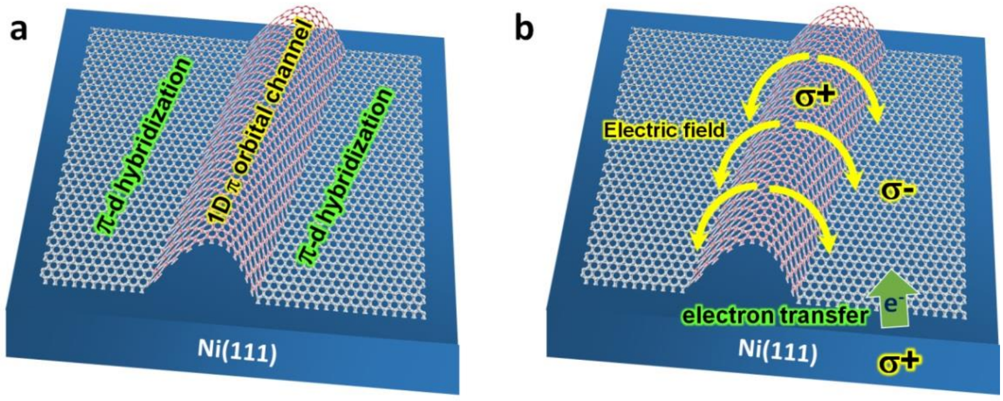

Supplementary Figure 11 | Illustrations of the mechanisms. a, 1D electron confinement in GNR and **b**, the electric field induced across a GNW due to electron transfer from Ni(111) to pEG.

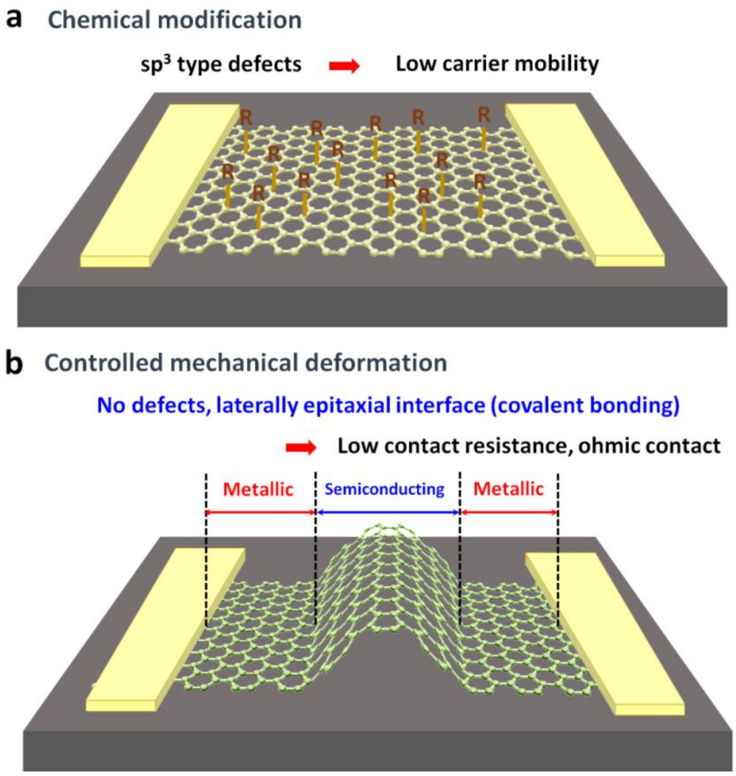

Supplementary Figure 12 | Schematic comparison of the chemical approach and controlled mechanical deformation. a. chemical modification. **b.** controlled mechanical deformation.

	**Method 1.** (circular shape assumption)	**Method 2.** (from height profile)	**Error**
**i**	1.76 nm	1.79 nm	+ 1.6 %
**ii**	2.35 nm	2.34 nm	- 0.4 %
**iii**	2.83 nm	2.99 nm	+ 5.6 %
**iv**	3.03 nm	3.06 nm	+ 0.9 %
**v**	3.04 nm	3.27 nm	+ 7.6 %
**vi**	3.70 nm	3.73 nm	+ 0.2 %

Supplementary Table 1| Arc length values for GNWs i – vi estimated by Method 1 and Method 2, respectively.

Supplementary Note 1. Epitaxial graphene (EG) growth and characterization

STM images obtained near the edge of the EG (Supplementary Fig. 1a) show both the EG and regions outside of the EG. The surface of the EG was very clean and smooth, whereas the surface observed outside the EG area was rough. The graphene lattice was clearly confirmed, as shown in Supplementary Fig. 2b. No Moiré patterns were observed on most of the EG area because of the lattice matching between the EG and Ni(111) surface[1,2]. Outside the EG, line structures of Ni_2C and many contaminants were confirmed (Supplementary Fig. 1c), and the surface structure of the Ni_2C in Supplementary Fig. 1d was almost the same as the [$(39)^{1/2}$ R16.1° × $(39)^{1/2}$ R16.1°] Ni_2C reported in the literature[2]. It is noteworthy that only a few EG surfaces showed Moiré patterns, which were induced mainly by rotated orientation (Supplementary Fig 2a, b)[1,2]. In the atomically resolved STM image in Supplementary Fig 2b, the graphene lattice and Moiré patterns were simultaneously confirmed. Moiré periodicity (|**M**|) and the angle (ρ) between the graphene unit vector ($\mathbf{a_{gr}}$) and Moiré vector (**M**) were confirmed as 3.12 nm and +9° (or –9°), respectively. The rotation angle ($R\theta$) of the EG with respect to the Ni($1\bar{1}0$) lattice can be estimated as $R\theta$ = 3.68 ° or 26.7 ° by Supplementary Equation 1 [3] on the assumption that lattice contraction (Δa_{Gr}) in EG is zero (k_S : reciprocal of the lattice unit vector of the substrate).

$$\rho = \tan^{-1}\frac{(|k_{S1}|\times\sin\theta)}{\left(\left|\frac{2\pi}{a_{Gr1}\times\left(1-\frac{\Delta a_{Gr1}}{a_{Gr1}}\right)}\right|\times|k_{S1}|\cos\theta\right)} \quad (1)$$

$$|\mathbf{M}| = 2\pi\sqrt{\left(\left|\frac{2\pi}{a_{Gr}\times\left(1-\frac{\Delta a_{Gr}}{a_{Gr}}\right)}\right|\times\sin\theta\right)^2 + (|k'_{Gr1}|\times\cos\theta - |k'_{S1}|)^2}^{\,-1} \quad (2)$$

Then, |**M**| could be calculated as 3.31 (for $R\theta$ = 3.68°) or 0.54 nm (for $R\theta$ = 26.7°) by using Supplementary Equation 2. We therefore concluded that the Moiré pattern in Supplementary Fig. 2 had R3.68 ° orientation.

Supplementary Note 2. Mechanism of formation of GNWs

As shown in Fig. 2a and b, GNWs formed at the step edges of the underlying Ni(111) surface or emerged from the kink of the step edges. The implication is that the underlying Ni(111) substrate played a critical role in the formation of the GNWs. Pan et al. have reported

step-induced graphene wrinkles and created steps with a height of 20–30 nm on the surface of Cu foil by a mechanical process[4]. These steps resulted in the formation of many wrinkles with a mean width of 15.4 nm. Our results can also be explained by the "step-induced wrinkle" mechanism, because atomic-scale steps exist intrinsically on the surface. During the cooling process in EG synthesis, the difference in thermal expansion between the EG and Ni surface induces a compressive strain in the EG (Supplementary Fig. 4 (left)). Compared to the EG on the flat terrace of the Ni substrate, the EG at the step edge does not interact strongly with the substrate; therefore, due to the strain, it can be folded only on the step edge (Supplementary Fig. 4 (right)). In general, graphene wrinkles on the terrace of an underlying substrate have been simply understood to result from substrate contraction due to differences of thermal expansion. However, most of the GNWs, even on the terrace, emerged from the kink of the step edge. This process is usually not observed on a bare Ni(111) surface. Although the mechanism of formation of GNWs on the Ni(111) terrace region has yet to be completely understood, we argue that step edges of Ni(111) play a critical role, even for the GNWs on the terrace region.

Supplementary Note 3. Estimation of arc lengths from widths and heights of GNWs

-Method 1. Estimation with height and width value base on the assumption of a circular shape

To compare our results with the energy gap in SWCNTs and graphene nanoribbons, we estimated the arc length (l) of the GNWs with height (h) and width (w) values measured by STM, because the length for electron confinement in the GNW was comparable to the circumference of SWCNTs and widths of graphene nanoribbons. For the calculation, we assumed that the GNW was part of a circle (Supplementary Fig. 6). By using Supplementary Equation 3 and a simple trigonometric function, we calculated the arc length for the GNWs (Fig. 3a). The ΔE_gs with respect to the calculated arc length are plotted in Fig. 3b. All calculated arc length values, l, are listed in Supplementary Table 1.

$$l = h^{-1}\left(h^2 + \left(\frac{w}{2}\right)^2\right) \times \sin^{-1}\left(\frac{hw}{h^2+\left(\frac{w}{2}\right)^2}\right) \tag{3}$$

-Method 2. Estimation from the height profile measured by STM

To confirm the reliability of Method 1, we estimated the arc length directly from the experimentally measured height profile by using the following Supplementary Equation 4.

$$l = \int_a^b \int \sqrt{(dx)^2 + (dy)^2}dx \quad \text{(for continuous function)}$$
$$= \sum_{i=1}^{n} \sqrt{(\Delta x)^2 + (\Delta y)^2} \quad \text{(for discrete data)} \qquad (4)$$

The calculated arc length values from height profile (Method 2) are additionally included with values estimated by Method 1 in Supplementary Table 1. The error between the values by two methods is confirmed as – 1 ~ 8 %, but in the cases of GNW ii and GNW iv having higher error values (5.6 % and 7.6 %), their arc lengths were overestimated due to the atomic protrusion or the noise on the height profile. Supplementary Figs. 8a,b show the height profiles of GNWs ii and v, respectively. While GWN b has a smooth height profile, the GNW e has more rough height profile, which might be originated not only by the atomic protrusion but also the noise during the measurement. This implies that the direct measurement from the experimentally measured height profile can provide a more relevant quantity, but some cases can provide an overestimated quantity such as GNWs ii and v. Nevertheless, we confirmed that the arc lengths obtained by our method (Method 1) based on the assumption that the GNW arc shape is part of a circle c is comparable with the experimental values obtained by the method 2, except some specific cases having higher roughness on their height profiles. Therefore, we believe that our assumption can be conveniently used for estimating the GNW arc length.

Supplementary References

1 Bao, W. *et al.* Controlled ripple texturing of suspended graphene and ultrathin graphite membranes. *Nat. Nanotechnol.* **4**, 562-566, (2009).

2 Lahiri, J. *et al.* Graphene growth on ni(111) by transformation of a surface carbide. *Nano Lett.* **11**, 518-522, (2011).

3 Pan, Z., Liu, N., Fu, L. & Liu, Z. Wrinkle engineering: A new approach to massive graphene nanoribbon arrays. *J. Am. Chem. Soc.* **133**, 17578-17581, (2011).

4 Saito, R., Dresselhaus, G. & Dresselhaus, M. S. *Physical properties of carbon nanotubes*. (Imperial College Press, 1998).
